# Conformational changes in α7 acetylcholine receptors underlying allosteric modulation by divalent cations

**DOI:** 10.1186/1471-2210-9-1

**Published:** 2009-01-13

**Authors:** James T McLaughlin, Sean C Barron, Jennifer A See, Robert L Rosenberg

**Affiliations:** 1 Department of Pharmacology, University of North Carolina at Chapel Hill, Chapel Hill, NC, 27599-7365, USA; 2 Department of Cell & Molecular Physiology, University of North Carolina at Chapel Hill, Chapel Hill, NC, 27599-7365, USA

## Abstract

Allosteric modulation of membrane receptors is a widespread mechanism by which endogenous and exogenous agents regulate receptor function. For example, several members of the nicotinic receptor family are modulated by physiological concentrations of extracellular calcium ions. In this paper, we examined conformational changes underlying this modulation and compare these with changes evoked by ACh. Two sets of residues in the α7 acetylcholine receptor extracellular domain were mutated to cysteine and analyzed by measuring the rates of modification by the thiol-specific reagent 2-aminoethylmethane thiosulfonate. Using Ba^2+ ^as a surrogate for Ca^2+^, we found a divalent-dependent decrease the modification rates of cysteine substitutions at M^37 ^and M^40^, residues at which rates were also slowed by ACh. In contrast, Ba^2+ ^had no significant effect at N^52^C, a residue where ACh increased the rate of modification. Thus divalent modulators cause some but not all of the conformational effects elicited by agonist. Cysteine substitution of either of two glutamates (E^44 ^or E^172^), thought to participate in the divalent cation binding site, caused a loss of allosteric modulation, yet Ba^2+ ^still had a significant effect on modification rates of these residues. In addition, the effect of Ba^2+ ^at these residues did not appear to be due to direct occlusion. Our data demonstrate that modulation by divalent cations involves substantial conformational changes in the receptor extracellular domain. Our evidence also suggests the modulation occurs via a binding site distinct from one which includes either (or both) of the conserved glutamates at E^44 ^or E^172^.

## Background

Allosteric modulation of membrane receptors is increasingly recognized as a common mechanism used to control cellular signal transduction [1,2]. In general, allosteric modulator binding causes changes in the response of the receptor to the "native ligand", presumably by altering the energetic barrier between resting and activated conformations. In most cases the modulator does not activate the target receptor in the absence of agonist. While there has been substantial progress in identifying the binding sites for many allosteric modulators (for example, [3]), the mechanisms by which modulators induce their effects remain poorly defined.

Some of the best examples of allosteric modulation involve members of the Cys-loop family of ligand-gated ion channels that includes nicotinic AChRs as well as the GABA_A_, glycine, and 5-hydroxytryptamine-3 receptors [4]. Cys-loop receptors transduce the energy of agonist binding into conformational changes that lead to channel opening [5]. All family members share a similar structure: they are transmembrane proteins assembled from five homologous or identical subunits. Each of these subunits is comprised of a large amino terminal extracellular domain (ECD), a large intracellular loop, and a four α-helix bundle forming a transmembrane domain (TMD). Recent studies aimed at identifying the structural basis for ligand gating have focused on the "transition zone" [6] a region of the receptor at the boundary between the ECD the TMD. The transition zone includes structural elements thought to link the TMD and the ligand binding site [7-9]. While the evidence for this linkage is preliminary, a number of experimental approaches have unequivocally mapped the site for ligand binding to the interface of adjacent subunit ECDs [5]. More recently, the crystal structures of ACh-binding proteins (AChBPs) from *Lymnea*, *Aplysia*, and *Bulinus *[10-12] have provided a structural context for these biochemical and functional studies. The AChBPs are soluble proteins that act as ACh buffers in invertebrates [13]; they share both sequence and functional homology to the ECD of Cys-loop receptors. Earlier this year another structure, that of a homologous bacterial ligand-gated ion channel, was added to the structural database of Cys-loop receptors [14]. These crystal structures have been used to develop and refine homology models of Cys-loop receptors [15,16]. Our goal is to use these refined models to test specific mechanistic hypotheses that attempt to explain the dynamics of both ligand-induced receptor activation and allosteric modulation [7-9,17].

Many neuronal nicotinic AChRs exhibit positive allosteric modulation by physiological concentrations of Ca^2+ ^[18,19]. In α7 nAChRs (but not other neuronal AChRs) Ba^2+ ^or Sr^2+ ^can elicit effects similar to Ca^2+ ^[18,20,21]. This modulation consists of an increase in both the efficacy and the potency of ACh. The functional effects of divalents are similar to those caused by an emerging class of nicotinic modulating drugs collectively referred to as PAMs (positive allosteric modulators; [22]). Thus one rationale for a mechanistic characterization of divalent modulation of α7 AChRs is to serve as a model for studies of drugs such as PAMs developed to elicit a similar effect.

Previous studies demonstrated that the modulation of α7 AChRs by divalent cations is independent of divalent cation permeation, suggesting that the binding site for modulation is extracellular [21]. In addition several studies have demonstrated the importance of conserved ECD glutamate residues (E^44 ^and E^172 ^in chick α7) in divalent cation modulation, and it has been suggested that these may form the allosteric modulation binding site [15,20,23]. In this paper, we tested the hypothesis that conformational changes evoked by divalent cation modulators of the α7 AChR are similar to those evoked by ACh. In addition, we examined whether E^44 ^and E^172 ^are required for divalent cation-evoked conformational changes. We found some similarities between Ba^2+ ^evoked conformational changes and those caused by ACh. Surprisingly, we also found that the effects Ba^2+ ^on modification rates did not require E^44 ^or E^172^, suggesting that these residues do not form the divalent cation binding site.

## Results

The substituted cysteine accessibility method (SCAM) is an established experimental approach to examine protein conformational dynamics [24]. We previously used this approach to scan regions of the chick α7 AChR and identified residues where the rates of thiol-specific modification by MTSEA were altered by ACh [23,25]. We consider two alternative mechanisms for ACh-dependent effects on modification rates. If the substituted cysteine is at a position that is part of the agonist binding site [5], then the effect of ACh could be due to steric occlusion. Alternatively, if the substituted cysteine is not near the binding site, then we infer that a change in modification rates is a result of conformational or electrostatic change induced by agonist-dependent activation. In this way, these residues serve as reporters of intramolecular changes during receptor activation.

**Figure 1:**
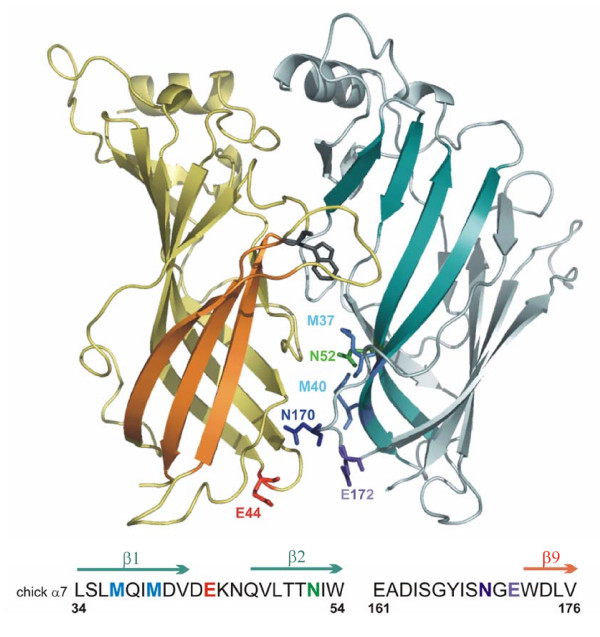
**A model of the α7 AChR extracellular domain**. Ribbon cartoon showing two of the five subunits viewed from the outside. In the subunit to the left of the central interface (yellow), the outer β sheet in is highlighted in orange, the transition zone E^44 ^residue is orange, and the W^148 ^residue is shown in gray to identify the ACh binding pocket (Zhong et. al., 1998). The subunit to the right shows a view of the inner sheet (teal), and other residues targeted in this study. The sequence surround mutants characterized in this study is shown beneath the cartoon: M^37^, M^40 ^cyan; N^52 ^green; N^170 ^blue; E^172 ^purple.

SCAM can be used in the same way to identify conformational changes caused by allosteric modulators. Figure 1 shows a representation of the region of the α7 ECD targeted in this study. A discrete region of the inner β sheet, including M^37^, M^40^, and N^52 ^was initially chosen to examine the effects of the divalent cation Ba^2+ ^on MTSEA modification rates. We also examined the effects of Ba^2+ ^at transition zone residues previously implicated in modulation by divalent cations, including E^44^, E^172^, as well as an adjacent position N^170^. All of the cysteine replacements at these residues have previously been shown to exhibit agonist-sensitive MTSEA modification rates, allowing us a basis for comparison for the effects of Ba^2+^.

For these studies we began by confirming the modulatory effects of Ba^2+ ^on ACh-dependent activation of our parental phenotype, α7 C^115^A/L^247^T. Wild-type α7 AChRs exhibit a complex positive modulation by divalent cations such as Ca^2+ ^or Ba^2+ ^that includes increases in both efficacy and potency [20]. In contrast, receptors with the L^247^T phenotype typically exhibit a simplified modulatory response consisting only of a 5- to 10-fold left shift in the ACh dose-response.

**Figure 2:**
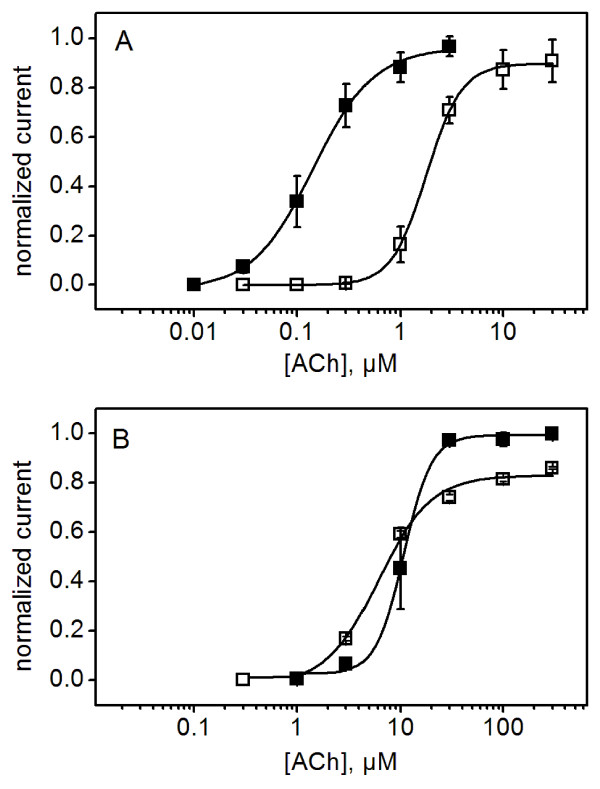
**Positive allosteric modulation by divalent cations requires E^44^**. ACh dose-response curves for the parental C^115^A/L^247^T (A) and the E^44^C mutant (B) in the absence (open squares) and presence (filled squares) of 10 mM BaCl_2_. Data are fitted to the Hill equation (solid lines). The positive allosteric modulation (leftward shift in the dose response curve) typically exhibited by α7 AChRs (A) is lost in the E^44^C mutant (B). Data are mean values (± SEM) from three determinations, normalized to the maximal value of the Hill equation fit of each data set. Hill coefficients for C^115^A/L^247^T (A): 2.5 ± 0.2 (open squares, -Ba^2+^), 1.9 ± 0.4 (filled squares, +Ba^2+^); and for the E^44^C mutant (B) 1.7 ± 0.2 (open squares, -Ba^2+^), 2.9 ± 0.7 (filled squares, +Ba^2+^).

Figure 2A shows the effect of 10 mM Ba^2+ ^on the α7 C^115^A/L^247^T receptor. There was a leftward shift in the dose-response curve corresponding to a ~10 fold decrease in EC_50 _(increase in potency). Of note, we do not see an effect of Ba^2+ ^on efficacy in the parental background. We suspect this is due to the higher gating constant of receptors with the L^247^T mutation. Figure 2B shows that modulation by Ba^2+ ^was eliminated in the E^44^C mutant, confirming that this conserved glutamate is required for Ba^2+ ^binding or allosteric coupling of Ba^2+ ^binding to ACh-dependent activation. This result is similar to the effect of an E^44^Q mutant described in wild-type and L^247^T α7 AChRs [20,21]. Table 1 provides a compilation of EC_50_'s, modulatory effects of 10 mM Ba^2+^, and the maximal responses of the mutants described in this report. Neither of the transition zone glutamate mutants (E^44^C and E^172^C) exhibited a positive modulation, while the N^52^C mutant displayed a high partial agonism by Ba^2+ ^in the absence of ACh.

**Table 1:** Effects of Ba^2+ ^on ACh evoked currents.

**mutant**	**Ach**	**EC_50_, μM**	**(n)**	**ACh I_max _μA**	**Ba^2+ ^efficacy^*a*^**
	*control*	*+ 10 mM Ba*^2+^			

C^115^A/L^247^T	1.8 ± 0.4	0.17 ± 0.02	4	6.9	0.1

M^37^C	2.0 ± 0.5	0.35 ± 0.12	4	0.92	0.2

M^40^C	7.7 ± 1.1	1.1 ± 0.1	5	3.0	0.1

N^52^C	2.6 ± 1.3	nd^*b*^	5	0.34	0.6

E^44^C	7.8 ± 0.4	10.1 ± 0.4	4	5.4	0.01

N^170^C^*c*^	13 ± 1.7	2.1 ± 0.4	6	1.7	0.03

E^172^C^*c*^	30 ± 2.7	50 ± 9.2	4	1.3	0.02

M^40^C/E^172^Q^*c*^	85 ± 6.2	134 ± 11	5	0.7	0.02

ACh dose-response measurements in the absence or presence of Ba^2+^. EC_50 _estimates are mean values ± SEM.

(*a*) Ba^2+ ^efficacy is the fraction of maximal ACh-evoked current that is evoked by 10 mM Ba^2+ ^in the absence of ACh. (*b*) nd: not determined because Ba^2+ ^was a strong partial agonist. (*c*) Mutants studied using co-expression with human RIC-3 (Halevi et al., 2002).

Previously, we measured the effects of ACh on reactivity of cysteine mutants in the inner β sheet of the chick α7 AChR [26]. Several residues (M^37^C, M^40^C, and N^52^C) exhibited a change MTSEA reaction rates in the presence of ACh. We interpret differences in modification rates in the absence or presence of ACh to reflect differences in the apparent accessibility of the introduced cysteine between the unliganded and liganded states. To test if these sites could also be used as reporters of allosteric modulator-induced conformational change we examined the effects of Ba^2+ ^on rates of MTSEA modification.

**Figure 3:**
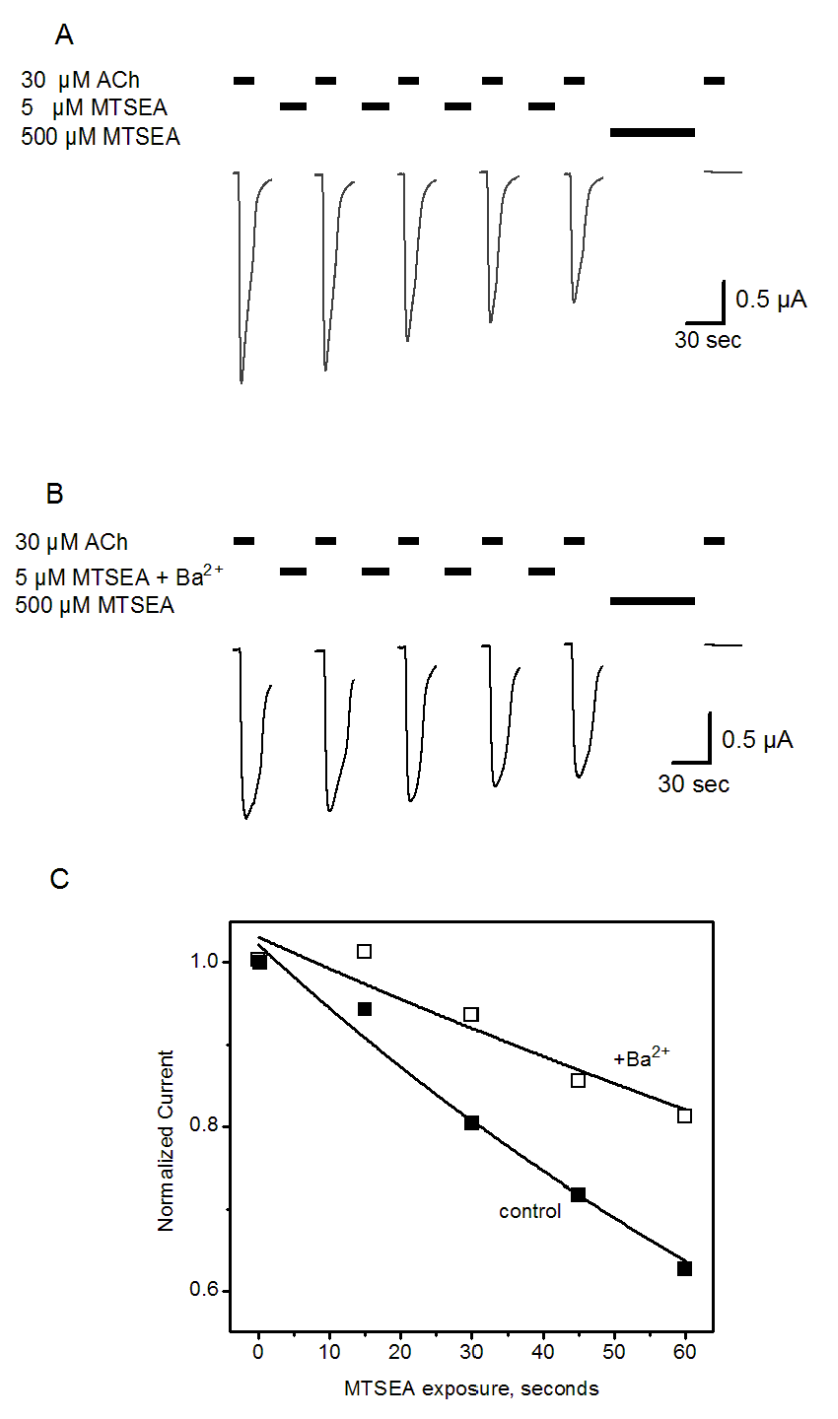
**Barium slows the rate of MTSEA modification at M^37^C**. Example of experimental paradigm used to assess Ba^2+ ^effects on modification rates. (A) Successive ACh-evoked current traces recorded before and after repeated exposures to MTSEA (5 μM, 15 seconds), showing a decrement in responses to 30 μM ACh. Endpoints of MTSEA modification are determined by prolonged application of 500 μM MTSEA (right). (B) The same protocol, including Ba^2+ ^pretreatment and co-application with MTSEA. Current traces are truncated in both (A) and (B) between consecutive MTSEA applications; in all cases the currents were allowed to return to baseline prior to the next application of MTSEA ± ACh. (C) Peak current amplitudes from (A) and (B) are normalized and plotted versus total MTSEA exposure time. Data from this single experiment (no error bars) are fitted to a single-exponential decay (solid line) to extract an apparent pseudo first-order rate constant. The pseudo first-order rate constants calculated in this experiment were 0.011 s^-1 ^and 0.0019 s^-1 ^for control (A) and +Ba^2+ ^(B) measurements, respectively. Second-order rate constants are calculated from these values (Figures 4–6; Table 2).

**Figure 4:**
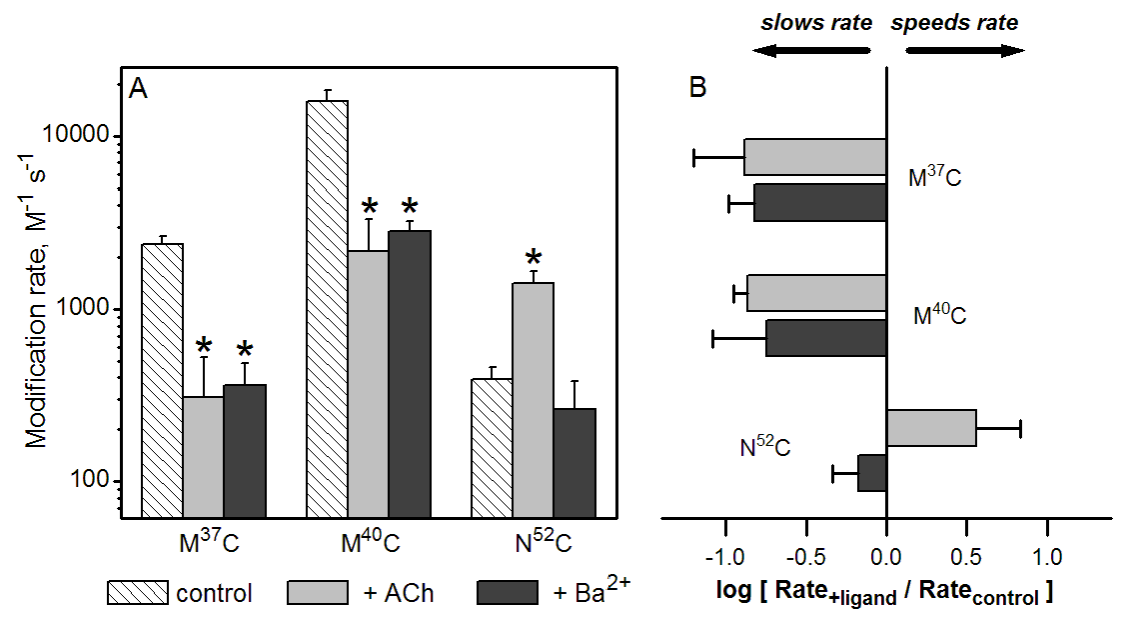
**Barium alters the rate of MTSEA modification at inner β sheet residues**. (A) Using the protocol described in Figure 3, we determined second-order rate constants for three reporter residues in the α7 AChR inner β sheet (M^37^C, M^40^C, and N^52^C). Mean values for second-order rate constants for modification by MTSEA alone (control), MTSEA + ACh, and MTSEA + Ba^2+ ^are shown. * Rate was significantly different from control (P < 0.05). (B) A plot of the ratios of second-order rate constants. Ba^2+ ^and ACh both slowed the rates of modification of M^37^C and M^40^C. At N^52^C, however, the rate of modification in the presence of Ba^2+ ^was not significantly different from control, while ACh accelerated the modification rate. See Table 2 for summary including (n) for each condition.

Figure 3 shows an example of the protocol used to measure the thiol modification rate of the receptors with the M^37^C mutation. ACh-evoked current amplitudes decreased following brief, repeated exposure to a limiting concentration of MTSEA (5 μM, 15 seconds). To ensure that the modification reactions were complete, all rate measurements included a final prolonged application of ~100-fold higher concentrations of MTSEA (Fig. 3A and 3B, right). Currents measured following this application represent the endpoint of the reaction between MTSEA and receptors. When the same protocol included Ba^2+ ^pretreatment and co-application with MTSEA (see Methods), the decreases in current amplitudes were slowed but the same endpoint was obtained (Fig. 3B). Normalized current amplitudes were plotted as a function of the cumulative time of exposure to MTSEA, and pseudo first-order rates were extracted from the single-exponential fits (Fig. 3C). We observe a significant decrease in the MTSEA modification rate for M^37^C (Fig. 4) in the presence of 10 mM Ba^2+^, demonstrating that this modulator caused changes in the conformation or electrostatic environment around the M^37^C side chain.

Using the protocol described in Figure 3, we determined second-order rate constants for modification of the three reporter residues in the inner β sheet (M^37^C, M^40^C, and N^52^C). Figure 4A shows mean values of the rate constants measured in the presence of MTSEA alone, MTSEA plus ACh, and MTSEA plus Ba^2+^. We observed significant decreases in reaction rates of both M^37^C and M^40^C in the presence of 10 mM Ba^2+^. The effects are quantitatively similar to those measured in the presence of ACh [26], consistent with the idea that Ba^2+ ^causes conformational changes similar to those induced by agonist in this region of the α7 AChR. To more directly compare the effects of ACh and Ba^2+ ^on reaction rates, we plot rate constants as ratios in Figure 4B. This figure highlights the differences in MTSEA rates under different conditions at these three positions. In contrast to M^37^C and M^40^C, we observed no significant effect of Ba^2+ ^on MTSEA modification rate of N^52^C. This result parallels our previous study in which we found the effect of ACh on MTSEA modification rate of N^52^C was also different from that of neighboring residues M^37^C and M^40^C. Collectively, these results suggest that divalent cations such as Ba^2+ ^act to promote some, but not all, of the conformational or electrostatic changes elicited by ACh. While ACh acts to stabilize the open state, Ba^2+ ^acts to stabilize a state (or states) that are energetic intermediates between closed and open channels.

**Figure 5:**
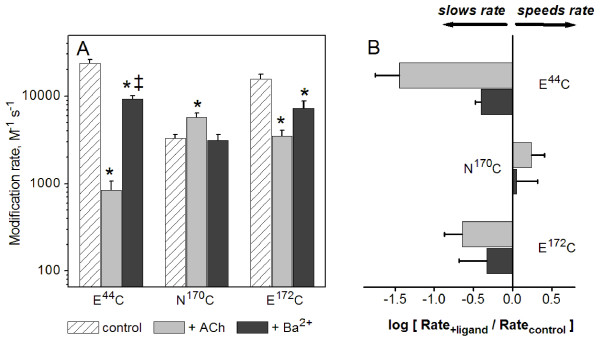
**Barium alters the rate of MTSEA modification at residues required for modulation by divalent cations**. Second-order rate constants were measured for three residues in the "transition zone" of the α7 AChR (E^44^C, N^170^C, and E^172^C). (A) Mean values for second-order rate constants for modification by MTSEA alone, MTSEA + ACh, and MTSEA + Ba^2+^. Ba^2+ ^caused a significant decrease in MTSEA modification rates of both E^44^C and E^172^C, despite the loss of divalent cation-dependent modulation. Ba^2+ ^did not have a significant effect on the modification rate of N^170^C, although ACh significantly increased the rate of modification of this residue. *Rate was significantly different from control (P < 0.05). ‡Rate was significantly different from that obtained in presence of ACh (P < 0.05). The plot of rate constant ratios (B) shows that the effect of Ba^2+ ^on the rate of modification of E^44^C was significantly less than the effect of ACh. See Table 2 for summary including (n) for each condition.

Modulation by divalent cations is known to require the conserved acidic residues at E^44 ^and E^172 ^(23, 24). We next tested whether Ba^2+ ^could cause changes in the rates of MTSEA modification at E^44^C, N^170^C, and E^172^C. Similar to residues in the inner β sheet, each of these mutants has been shown to be a reporter of conformational or electrostatic changes induced by ACh [23,25]. Figure 5A shows mean values of second order rate constants measured in the presence of MTSEA alone, MTSEA plus ACh, and MTSEA plus Ba^2+^. At N^170^C, a mutant that showed allosteric modulation (Table 1), the rate of MTSEA modification in the presence of Ba^2+ ^was the same as that measured in MTSEA alone, but was different from that measured in the presence of ACh. This observation suggests that conformational or electrostatic changes induced by modulators at this residue are distinct from those induced by ACh. Differences between the effects of ACh and Ba^2+ ^were most pronounced at E^44^C; at this residue, the modification rate was ~10 fold higher in the presence of Ba^2+ ^compared to that measured in the presence of ACh (Figure 5B). Surprisingly, despite the fact that both E^44^C and E^172^C show no positive allosteric modulation of ACh currents by Ba^2+ ^(Fig. 2, Table 2), both exhibited significant Ba^2+^-dependent decreases in MTSEA modification rate.

**Table 2:** Summary of MTSEA modification data.

**MUTANT**	**MTSEA**	**modification rate**	**× 10^3^, M^-1 ^S^-1 ^(n)**
	*control*	*+ ACh*	*+ Ba*^2+^

M^37^C	2.4 ± .03 (10)	0.30 ± 0.05 (4)	0.40 ± 0.02 (5)

M^40^C	16 ± 0.2 (13)	2.2 ± 0.4 (3)	2.8 ± 0.05 (7)

N^52^C	0.40 ± .08 (9)	1.4 ± 0.04 (7)	0.26 ± 0.02 (7)

E^44^C	23 ± 0.7 (4)	0.90 ± 0.05 (4)	9.3 ± 0.1(8)

N^170^C^*a*^	3.3 ± .05 (7)	5.7 ± 0.1 (7)	3.7 ± 0.06 (9)

E^172^C^*a*^	15 ± 0.4 (5)	3.5 ± 0.1 (5)	7.3 ± 0.3 (6)

M^40^C/E^172^Q^*a*^	14 ± 0.5 (5)	1.3 ± 0.1 (5)	2.0 ± 0.3 (6)

Second-order rate constants ± standard error for various conditions at each Cys mutant. (n), number of determinations.

(*a*) Mutants studied using co-expression with human RIC-3 (Halevi et al., 2002).

The hypothesized requirement for E^44 ^and E^172 ^in divalent cation modulation was based upon studies of charge neutralization mutants (E^44^Q, E^172^Q) in which modulation is lost. From these and other studies, both residues were proposed to be participants in a binding site which mediates the divalent cation allosterism [20,21]. The loss of Ba^2+ ^dependent modulation in E^44^C or E^172^C mutations (also charge neutralization mutations) is consistent with this proposal, but the effects of Ba^2+ ^on MTSEA modification rates are not. One possible explanation for these observations is that Cys replacements at E^44 ^and E^172 ^do not prevent Ba^2+ ^binding, but cause an uncoupling of binding and allosteric modulation. If Ba^2+ ^binds near E^44^C and E^172^C, the slowed modification rate at E^44^C or E^172^C would be explained by physical occlusion of the thiol side-chain by bound Ba^2+^. Alternatively, the glutamates could be a required component in the transduction pathway between Ba^2+ ^binding and receptor modulation, but are not direct participants in the binding site. In this case divalent cations bind at a different site and elicit conformational or electrostatic changes (detected as changes in E^44^C and E^172^C modification rates), but binding does not lead to modulation. To test this possibility we examined MTSEA modification rates at M^40^C α7 AChRs in which a second, charge-neutralizing mutation (E^172^Q) was introduced. We reasoned that if E^172 ^is required for binding of divalent cations, then the modification rate of M^40^C should be insensitive to Ba^2+^. If, however, Ba^2+ ^binds to the receptor and causes conformational changes, despite the mutation at E^172^, this would be reflected by changes in the rate of modification of M^40^C.

**Figure 6:**
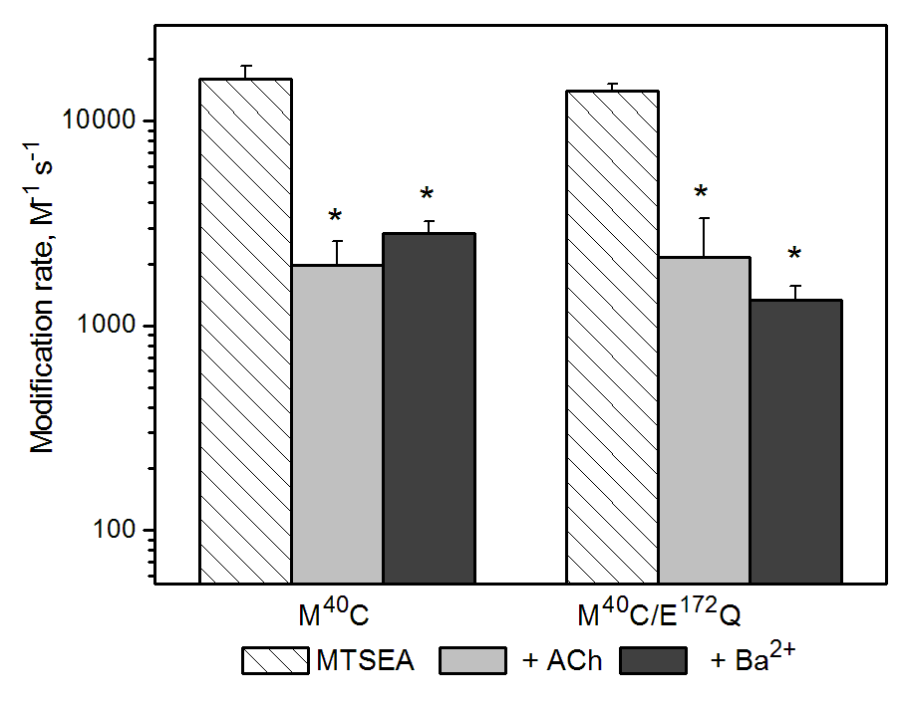
**Charge neutralization at E^172 ^does not alter the rate of modification M^40^C by MTSEA**. Mean values for second-order modification rate constants for M^40^C (left, data from Fig. 3) compared to those obtained in receptors containing the E^172^Q mutation (M^40^C/E^172^Q). *Rates were significantly different from control (P < 0.05). See Table 2 for summary, including (n) for each condition.

We measured dose-response relationships for the M^40^C/E^172^Q double mutant in the absence and presence of 10 mM Ba^2+ ^and confirmed that it was not positively modulated by Ba^2+ ^(Table 1). When the rates of MTSEA modification of the M^40^C in this background were measured, we found that Ba^2+ ^caused a significant slowing of MTSEA modification rate (Figure 6). The modification rates of M^40^C were independent of the E^172 ^mutation. This result suggests that the binding site for Ba^2+ ^modulation is somewhere other than a site which includes E^44 ^and E^172 ^in the α7 AChR transition zone [15].

## Discussion

The mechanisms of protein allosterism have been the subject of exhaustive modeling and model refinement since studies of Monod, Wyman and Changeux [27] and those of Koshland and colleagues [28]. In the nicotinic receptors, several different types of allosteric behavior have been described. The concerted, or MWC model, for example, refers to the allosteric effect of ligand binding on channel opening; this model suggested that binding of multiple agonists acted in concert to yield their "at a distance" effect. Experimental tests of the MWC model with combinations of agonists and antagonists suggest that a stepwise process more accurately describes the activation process [29,30].

Another type of allosterism seen in some nicotinic receptors is the positive allosteric modulation by divalents such as Ca^2+ ^or Ba^2+ ^[18,20]. For this type of allosterism, a fundamental question is whether the modulation alters the conformational "pathway" from closed to open states or simply modifies the kinetics of an agonist-dependent closed to open transition. Few studies have attempted to address this question, but a recent report does examine the conformational effects of positive allosteric modulators (benzodiazepines) in GABA_A _receptors [31]. This study used SCAM to show that a prominent effect of benzodiazepines is to increase the access of GABA to its binding site, reducing the energetic barrier to the initial step in receptor activation, GABA association. Divalent cation effects on α7 AChRs provide a similar paradigm in which to examine the conformational changes evoked by allosteric modulators.

In an earlier report we described an α7 AChR mutant with a pair of cysteine substitutions positioned to introduce a disulfide bond in the outer β sheet [26]. In our parental background this mutant was fully activated by divalent cations in the absence of ACh, but when expressed in an α7 AChR without the L^247^T mutation it required both ACh and divalent cations for channel activation. If we assume that activation of this mutant occurs as a result of Ba^2+ ^interaction with the divalent cation allosteric site, then it suggests that Ba^2+ ^and ACh promote two overlapping but distinct sets conformational changes. The experiments presented in this report provide further evidence that conformational effects of divalent cations are similar to those of elicited by ACh. Thus the simplest interpretation of our data is that divalent cations act by enhancing transitions in an ACh-dependent activation pathway without substantial effect on the final closed to open transition.

Le Novere and colleagues [15] proposed a model for a divalent cation binding site that was based on earlier experiments, homology between the α7 AChR ECD and the *Lymnea *AChBP, and the known database of divalent cation binding proteins [10,20]. The focus of this model was a cluster of 4 negatively charged residues in the transition zone: D^41^, D^43^, E^44^, and E^172^. Mutational analysis suggested that the glutamate residues were critical, since charge neutralization at either of the aspartate residues had only modest effects on divalent modulation. While the geometry of these residues in models of the ECD is consistent with their proposed model, our results with Ba^2+^-induced conformational changes are not. A mutation of either E^44 ^or E^172 ^to cysteine eliminates the modulation, but not the conformational changes associated with Ba^2+^modulation. This strongly suggests that the allosteric effects of Ba^2+ ^are "transmitted" in a conformational pathway that requires these glutamates for some role other than divalent cation binding. It is unlikely that the Cys substitution is able to act as a functional substitute for Glu in a divalent cation site: a survey of all known Ca^2+ ^binding sites found that Cys was never a contributor to a Ca^2+ ^co-ordination site, while it often plays this role in Zn^2+ ^binding sites [32]. Other possible candidates for a divalent cation modulation site in the α7 ECD include acidic residues in β6 and β8, which may combine with neighboring carbonyl groups to form a site for divalent cation binding. Alternatively, the recent work of Horn and colleagues [33] has demonstrated that aromatic residues may provide the negative electrostatic environment required for formation of a physiologically relevant divalent cation binding site through the π-cation-type interactions. This is the same structural motif that has been shown to provide the negative electrostatic environment in the cholinergic agonist binding site [34].

## Methods

### Reagents

MTSEA (2-aminoethylmethane thiosulfonate) was obtained from Toronto Research Chemicals (Toronto, Canada). Gentamicin was from Invitrogen (Carlsbad, CA). All other reagents were obtained from Sigma-Aldrich (St. Louis, MO).

### Site-directed mutagenesis

A cDNA clone of the chick α7 receptor containing two mutations (C^115^A, L^247^T) was used as the parental phenotype for mutations described in this study. We mutated the lone unpaired cysteine in the extracellular domain (C^115^) to alanine to allow for a more straightforward interpretation of thiol modification experiments. We observed no functional effect of this mutation on receptor expression or ACh response. We included the mutation of leucine 247 in the M2 transmembrane domain (L^247^T; L9'T) because of its large current amplitudes and non-desensitizing kinetics compared to wild-type α7 receptors [35]. These receptors exhibit a higher "gating constant" than wild-type α7 AChRs [36], suggesting that the closed-to-open equilibrium of liganded C^115^A/L^247^T receptors favors the open state. Mutation at the L^247 ^position enhanced the ability to measure modification rates for cysteine replacements in which the ACh-evoked current amplitudes are attenuated [37]. In preliminary experiments, modification rates of cysteines introduced into wild type α7 AChRs were similar to those in L^247^T-containing receptors, suggesting that conformational changes in the ECD of L^247^T-containing receptors are similar to those in wild-type receptors (not shown). All mutations were introduced by site-directed mutagenesis using the QuikChange method (Agilent Technologies, La Jolla, CA) as described previously [21], and were confirmed by DNA sequencing.

### Xenopus oocyte maintenance and expression

cRNA was prepared using the T7 RNA polymerase and mMessage mMachine kit as described by the manufacturer (Applied Biosystems, Austin, TX). Oocytes were surgically removed and prepared from female *Xenopus laevis *in accordance with UNC Institutional Animal Care and Use Committee guidelines. Oocytes were injected with 20 ng of cRNA and incubated at 18°C in ND96 (96 mM NaCl, 2 mM KCl, 1 mM MgCl_2_, 1.8 mM CaCl_2_, 5 mM Na pyruvate, 50 μg/ml gentamicin, 5 mM HEPES, pH 7.5) for 2–5 days before use. For some mutants we co-injected cRNA encoding the human RIC-3 [38], a protein shown to enhance expression of α7 AChRs in both mammalian cells and oocytes [39]. This co-injection (at a 1:1 ratio, 20 ng per oocyte) enhanced maximal current responses without significant effect on ACh EC_50 _(not shown).

### Data collection and analysis

Oocytes were superfused in normal extracellular solution containing a reduced Ca^2+ ^concentration (ESLC; 96 mM NaCl, 2 mM KCl, 1 mM MgCl_2_, 0.1 mM CaCl_2_, and 10 mM HEPES, pH 7.5). This solution minimized Ca^2+ ^influx and eliminated Ca^2+^-activated chloride currents. Two-electrode voltage clamp was performed with a GeneClamp 500B controlled by pCLAMP8 software (Molecular Devices, Sunnyvale, CA). Electrodes were filled with 3 M KCl contacting Ag-AgCl wires and had resistances of 0.5 to 2.0 MΩ. Currents were recorded at a constant holding potential of -60 mV. Currents were low pass filtered at 50 Hz and sampled at 100 Hz. Agonist dose-response curves were obtained as described previously [21], and data were fit to the Hill equation using Origin software (Microcal Software, Northampton, MA).

### Expression and modification rates

Each mutant was initially screened for functional expression over a range of ACh concentrations to generate a dose-response relationship and determine its EC_50_. To test for reactivity of introduced free thiols, we compared responses of each mutant to an ~EC_50 _ACh dose before and after exposure to high concentrations of MTSEA (0.5 – 1.0 mM) applied by continuous flow for 30 to 60 seconds. MTSEA was prepared daily in distilled water and stored on ice. Stock solution was diluted to the appropriate working concentration in ESLC immediately before each application. Rates were measured by determining a limiting dose of MTSEA (0.1–100 μM), then exposing oocytes to these low concentrations of MTSEA repeatedly for 15–30 seconds followed by a challenge with an ~EC_50 _concentration of ACh. The limiting dose, yielding 20–40% of the maximal MTSEA effect, was identified for each mutant. To measure the effect of ACh on modification rates we used the same protocol but included an ~EC_110 _ACh dose with the applied MTSEA. To measure the effect of Ba^2+ ^on modification rates we pre-applied 10 mM Ba^2+ ^for 30 seconds prior to co-application of 10 mM Ba^2+ ^plus MTSEA. This concentration of Ba^2+ ^is equivalent to an approximate EC_110 _for the modulatory effects in both parental and mutant AChRs. Kinetic data were analyzed as described previously [40]; rate data were fit to a single exponential to extract a pseudo-first order rate constant; this was divided by the MTSEA concentration used to determine the second order rate constant for thiol modification.

### Statistical Analysis

Statistical analysis of EC_50 _values and second-order rate constants was conducted using a one-way analysis of variance, followed by a post hoc Tukey test. P values of < 0.05 were interpreted to indicate significant differences.

### Structural models of α7

A model of the chick α7 nicotinic receptor extracellular domain, based on the coordinates of the *Lymnea *ACh Binding Protein [10] was constructed as described previously [23,26]. Images of the model were generated with Pymol (DeLano Scientific, South San Francisco, CA).

## Abbreviations

ACh: acetylcholine chloride; AChR: acetylcholine receptor; AChBP: acetylcholine binding protein; ECD: acetylcholine receptor extracellular domain; ESLC: extracellular solution, low calcium; GABA: gamma-amino butyric acid; MTSEA: 2-aminoethylmethane thiosulfonate; MWC: Monod-Wyman-Changeux model of allosterism; SCAM: substituted cysteine accessibility method; TMD: acetylcholine receptor transmembrane domain

## Authors' contributions

JTM designed and conducted experiments, performed data analysis, wrote and edited the manuscript. SCB designed and conducted experiments, performed data analysis, and edited the manuscript. JAS designed and conducted experiments, performed data analysis, and edited the manuscript. RLR designed experiments, performed data analysis, wrote and edited the manuscript.
